# Basic taste processing recruits bilateral anteroventral and middle dorsal insulae: An activation likelihood estimation meta‐analysis of fMRI studies

**DOI:** 10.1002/brb3.655

**Published:** 2017-03-10

**Authors:** Andy Wai Kan Yeung, Tazuko K. Goto, Wai Keung Leung

**Affiliations:** ^1^ Oral and Maxillofacial Radiology, Applied Oral SciencesFaculty of DentistryThe University of Hong KongHong KongChina; ^2^ Department of Oral and Maxillofacial RadiologyTokyo Dental CollegeMisakichoChiyoda‐kuTokyoJapan; ^3^ Periodontology, Faculty of DentistryThe University of Hong KongHong KongChina

**Keywords:** connectivity, functional magnetic resonance imaging, insula, meta‐analysis, neuroimaging, taste

## Abstract

**Background and Purpose:**

Numerous task‐based functional magnetic resonance imaging (fMRI) studies have reported the locations of basic taste representations in the human brain, but they usually employed a limited number of subjects (<20) with different methodologies and stimuli. Moreover, the reported brain regions were sometimes inconsistent. Thus, we aimed at performing a meta‐analysis of the published data to identify locations consistently activated across studies, and performed a connectivity analysis to reveal how these taste processing regions connect with other brain regions.

**Materials and Methods:**

A meta‐analysis was performed based on 34 experiments, with 238 total participants in 16 studies, to establish the activation likelihood estimation (ALE) of taste‐mediated regional activation. Meta‐analytic connectivity modeling (MACM) and data stored in BrainMap database were employed to reveal the functional connectivity of the regions identified by ALE with other brain regions, across all types of experiments that caused activation among healthy subjects.

**Results:**

ALE identified nine activated clusters in bilateral anteroventral and middle dorsal insulae, bilateral thalamus and caudate, bilateral pre‐/postcentral gyrus, and right hippocampus. The concurrence between studies was moderate, with at best 38% of experiments contributed to the significant clusters activated by taste stimulation. Sweet taste was the predominant contributing taste. MACM revealed that at least 50% of the nine clusters coactivated with the middle cingulate cortex, medial frontal gyrus, inferior parietal lobule, and putamen.

**Conclusion:**

Results suggested that fMRI studies have reported reproducible patterns of activations across studies. The basic taste stimulations resulted in activations in a mostly bilateral network. Moreover, they were connected with cognitive and emotional relevant brain regions.

## 1 Introduction

Taste is one of the most crucial basic senses that empowers humans to evaluate what foods to ingest for survival (i.e., nutrient absorption vs. potential contamination or toxicity) and enjoyment/reward (Breslin, 2013). Upon stimulation of taste receptors, neural signals are generated and relayed to the primary taste cortex, which then mediates the more complex perception and behavior pertaining to taste sense integrations and associations. Examples of such associations include phantom tastes (Henkin, Levy, & Lin, 2000), taste memory (Levy, Henkin, Lin, Finley, & Schellinger, 1999), semantic grounding of taste words (Barrós‐Loscertales et al., 2012), synesthesia involving tastes (Jones et al., 2011), taste enhancement by additives (Goto et al., 2016), taste inference related to viewing food‐imitating products (Basso et al., 2014), and visual food cues (van der Laan, De Ridder, Viergever, & Smeets, 2011). Understanding the mechanisms behind these associations will be difficult without first mapping out the brain regions important to basic taste sensation.

Past neuroimaging studies have investigated the neural correlates of various aspects of taste perception and eating behavior in the brains of healthy people, but they employed different methodologies and tastants, and utilized relatively small sample sizes (e.g., <20) that reduce their reliability (Raemaekers et al., 2007). Furthermore, sometimes the reported locations showing activation were different between studies. Hence, a meta‐analysis of these papers is necessary as it pools data collected with similar parameters to identify locations with a consistent response across studies (Eickhoff et al., 2009). The activation likelihood estimation (ALE) is a commonly used approach to achieve this (Eickhoff, Bzdok, Laird, Kurth, & Fox, 2012; Eickhoff et al., 2009, 2011; Laird, Fox, et al., 2005; Turkeltaub, Eden, Jones, & Zeffiro, 2002; Turkeltaub et al., 2012). It has already been used in neuroimaging meta‐analyses regarding taste perception (Kurth, Zilles, Fox, Laird, & Eickhoff, 2010; Veldhuizen et al., 2011) and viewing of food pictures (van der Laan et al., 2011; van Meer, van der Laan, Adan, Viergever, & Smeets, 2015).

Although there were already meta‐analyses of chemosensory perception of taste, this study was conducted to address four novel aspects. First, we only included data from reports on whole‐brain analyses. Second, we utilized the newly recommended statistical approach for ALE, namely the cluster‐level family‐wise error (FWE) correction, which should have increased sensitivity, a better control for false‐positive findings and excessive contributions by individual studies (Eickhoff et al., 2016). Third, for each significant basic taste‐activated brain cluster identified in the meta‐analysis, we also identified the types of tastes that contributed to its activation. Fourth, we performed meta‐analytic connectivity modeling (MACM) which, using data across studies stored in the BrainMap database, investigates the functional connectivity of the activated regions identified in the ALE with other brain regions (Fox & Lancaster, 2002; Fox et al., 2005; Laird, Lancaster, & Fox, 2005; Laird et al., 2011). To the best of our knowledge, this is the first study to perform connectivity analysis for meta‐analytic data of taste processing.

Therefore, the first purpose of this study was to revisit fMRI meta‐analysis of taste processing incorporating new data from recent task‐based fMRI studies and new statistical guidelines. This will produce a brain map showing consistent taste‐related activations across individual studies. The second purpose was to use MACM to reveal the patterns of connectivity between the identified taste processing regions and other brain regions. We hypothesized that the results would show significant clusters in regions frequently reported to activate upon basic taste stimulations, such as the bilateral thalamus and insula.

## 2 Materials and Methods

### 2.1 Literature search and selection criteria

**Figure 1:**
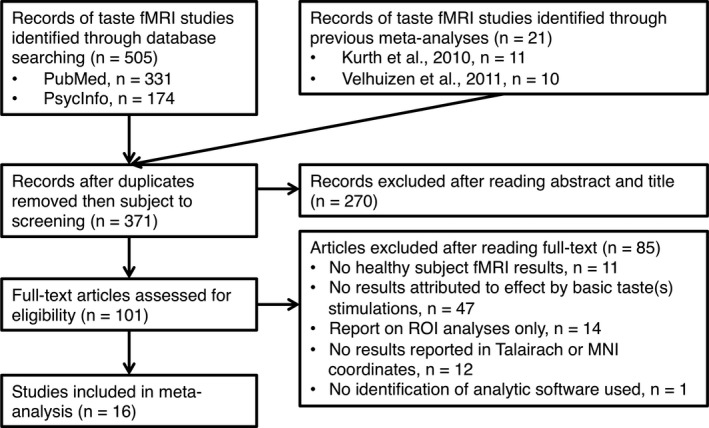
Flowchart of the review process. The number of publications (n) in each stage is labeled

PubMed and PsycInfo were searched (van der Laan et al., 2011; Tang, Fellows, Small, & Dagher, 2012; Veldhuizen et al., 2011) to identify human taste functional magnetic resonance imaging (fMRI) studies indexed until May 2016. The articles must contain the keywords (“functional magnetic resonance imaging” OR “MRI” OR “BOLD”) AND (“taste” OR “gustatory” OR “gustation” OR “tastants” OR “flavor”) in their title or abstract (Veldhuizen et al., 2011). “BOLD” stands for blood oxygenation level dependent, as fMRI studies typically detect BOLD signals. In addition, previous relevant meta‐analyses were identified (Kurth et al., 2010; Veldhuizen et al., 2011) and their selected articles entered our screening process. Studies employing positron emission tomography (PET) were not considered because PET has a lower spatial and temporal resolution than fMRI and thus the reported brain responses may not compare well (van Meer et al., 2015; Molenberghs, Johnson, Henry, & Mattingley, 2016; Sawyer, 2011). The database search revealed more than 500 studies (Figure 1), of which 371 records were unique and subsequently screened. As a first step, titles and abstracts were manually screened for their suitability. We searched for studies that were written in English and published in peer‐reviewed journals, employed healthy adult participants, and used liquid stimuli consisting of only basic tastes without odor or food components. We excluded food components because they might have a different texture from a control solution, might be odorous, or could trigger participants to recall their daily eating experiences (i.e., activate memory systems). Each of these could confound the brain activation attributable to chemosensory perception of taste and thus cause false positives. After this step, 101 records remained.

In the next step, the full text of the remaining 101 records was further evaluated based on an extra ordered set of five inclusion criteria listed below:

1. Reported results from healthy (i.e., systemic disease free) participants.
2. Reported results based on brain activations by taste stimuli (e.g., correlational analyses between brain response and behavioral scores were thus excluded).
3. Reported results based on whole‐brain analysis. ROI analyses results were excluded because they would bias the outcome of meta‐analyses by ALE (Eickhoff et al., 2009; Laird, Fox, et al., 2005; Turkeltaub et al., 2012). To supplement the main meta‐analysis, an additional meta‐analysis was conducted for 14 ROI studies that fulfilled the other four inclusion criteria.
4. Reported results in standardized stereotaxic space, that is, Montreal Neurological Institute (MNI) or Talairach spaces.
5. Reported the software used for processing and analyzing fMRI data.

Records needed to meet with all five criteria in order to be included in the meta‐analysis. Report of participants’ body mass index (BMI) was initially considered as an inclusion criterion, but was rejected because many publications did not report on this; in our final inclusion of 16 studies, only five had reported on BMI, while another one only noted that they had screened for BMI (see 3.1 in the *Results* section). Thus, 85 publications did not enter the meta‐analysis (Supplementary File 1). Since our selection criteria were different from those of Veldhuizen et al. (2011) and Kurth et al. (2010), this screening process eventually removed some of their primary studies while adding some new studies. The entire screening process yielded 16 publications for the meta‐analysis. The coordinates of activation clusters were extracted and those reported in Talairach space were converted to MNI coordinates by Lancaster transform (Lancaster et al., 2007). Subsequently, all MNI coordinates were entered into analyses together with the number of subjects from each experiment.

### 2.2 Activation likelihood estimation

To identify regions of consistent activation, we performed an ALE meta‐analysis. It produces a statistical parametric map, assigning an ALE value for each voxel that indicates the consistency of its activation across studies (Eickhoff et al., 2009, 2012; Turkeltaub et al., 2012). A voxel would have a higher ALE value if more studies reported activated peaks in or close to it.

The BrainMap GingerALE 2.3.6 program (Research Imaging Institute, 2016) was used to conduct the analysis. The computations were based on the revised ALE approach for coordinate‐based meta‐analysis of neuroimaging data that have been described in detail (Eickhoff et al., 2009, 2012; Turkeltaub et al., 2012). The standardized procedures are also found in the GingerALE user manual (Research Imaging Institute, 2013). In short, a map of MNI space was created for each entered study. Within the map, each voxel had a modeled activation (MA) score that reflected the probability of an activation being located there (Eickhoff et al., 2012). This was modeled as a three‐dimensional normal probability distribution centered at the input coordinates. Finally, the MA maps for all studies were unified on a voxel‐by‐voxel basis to calculate an ALE value for each voxel.

On the map of ALE values, a *p* value was calculated for each voxel based on the probability of observing an ALE value higher than the current value under the null‐distribution. This was achieved by randomly relocating ALE values across the volume, that is, via random permutation. In this study, the *p* values were generated by 5,000 permutations (Engelmann et al., 2012; Laird, Fox, et al., 2005; Laird, et al., 2010; Witt, Laird, & Meyerand, 2008). Clusters were considered active if the cluster‐level FWE was *p *<* *.05 after an initial cluster‐forming threshold of uncorrected *p *<* *.001 (Eickhoff et al., 2016). For this cluster‐level thresholding approach to ALE meta‐analysis, a minimum of 17 experiments should be incorporated into an independent meta‐analysis to control for the excessive influence from any single experiment (Eickhoff et al., 2016).

In addition, we recorded the percentage of contributing experiments and the types of tastes involved for each cluster reported from the meta‐analysis to help illustrate the contributions from each basic taste.

### 2.3 Visualization of meta‐analytic results

The thresholded ALE maps were overlaid onto the anatomical template, Colin27_T1_seg_MNI.nii (Holmes et al., 1998), provided on the GingerALE website. Visualization was carried out in Mango 3.8 (Research Imaging Institute and UTHSCSA, 2016). Local maxima of activation clusters were anatomically labeled with visual reference to an anatomical atlas (Mai, Majtanik, & Paxinos, 2016) and cross‐referenced with the MNI atlas provided by Mango.

### 2.4 Meta‐analytic connectivity modeling (MACM)

MACM was used to identify functional coactivation patterns between the significant clusters observed using ALE and other brain regions. Briefly, we created one volume of interest (VOI) for each significant cluster. The coactivation pattern was analyzed with neuroimaging data stored in the freely available BrainMap database (www.brainmap.org) (Laird et al., 2011). This approach allowed us to identify areas consistently coactivated with each VOI across all experiments indexed within the database. The database was accessed via BrainMap Sleuth 2.4.1b software (Fox & Lancaster, 2002; Fox et al., 2005; Laird, Lancaster, et al. 2005). At the time of the search, the database contained 2,994 papers reporting 14,720 experiments with 62,902 participants. Separate searches were performed for each VOI, and we limited papers to those that reported activation mapping of healthy subjects only. These coactivation data were transferred to GingerALE to perform independent ALE computations as described for the meta‐analysis above.

## 3 Results

### 3.1 Study and participant profiles

The data from the 16 studies included in the meta‐analysis involved 295 clusters of coordinates from 34 experiments utilizing 238 participants (105 males, 126 females, 7 unidentified) (Table 1). Each study enrolled 3–24 participants. Participants in eight studies were predominantly right‐handed, while eight studies did not report handedness. Participants were mainly 20–40 years old. Their fasting time before brain scanning ranged from 2–12 h. Nine studies used SPM for processing data, six used AFNI, and one used MEDx. One AFNI study (Green & Murphy, 2012) involved the use of FMRIB Software Library (FSL, another software program). Participants in 13 studies needed to swallow the taste sample liquids (Avery et al., 2015; Bender, Veldhuizen, Meltzer, Gitelman, & Small, 2009; Cerf‐Ducastel, Haase, & Murphy, 2012; Eldeghaidy et al., 2011; Green, Jacobson, Haase, & Murphy, 2013; Green & Murphy, 2012; Haase, Cerf‐Ducastel, Buracas, & Murphy, 2007; Haase, Cerf‐Ducastel, & Murphy, 2009; McCabe & Rolls, 2007; O'Doherty, Rolls, Francis, Bowtell, & McGlone, 2001; Small et al., 2003; Veldhuizen, Bender, Constable, & Small, 2007; Veldhuizen, Nachtigal, Teulings, Gitelman, & Small, 2010), while those in the remaining three studies did not (Kami et al., 2008; Nakamura et al., 2011, 2012).

**Table 1:** Studies included in the meta‐analysis of this study

Study	*n* a	Handed‐ness^b^	Mean age ± SD (range)	Fast time	Contrasts (stimuli)^c^	Foci	Statistical correction^d^	Software^e^
O'Doherty et al. (2001)	7 (gender unidentified)	NA	NA	NA	Glucose – tasteless; sodium chloride – tasteless	24	Uncorrected *p *<* *.01 + k > 3	MEDx
Small et al. (2003)	9 (3M, 6F)	R	24 ± NA (NA)	NA	High (sucrose/quinine sulfate) – low (sucrose/quinine sulfate); Sucrose – tasteless; Quinine sulfate – tasteless	12	FWE	SPM
Haase et al. (2007)	18 (9M, 9F)	NA	20.7 ± 1.0 (19–22)	NA	Sucrose – tasteless	14	Monte Carlo	AFNI
McCabe and Rolls (2007)	12 (6M, 6F)	NA	NA	NA	(MSG + IMP) – tasteless; NaCl – tasteless	4	FWE	SPM
Veldhuizen et al. (2007)	14 (3M, 11F)	R	26.2 ± 3.0 (NA)	NA	Taste (sucrose/ NaCl/ citric acid) – tasteless	7	Uncorrected *p *<* *.001	SPM
Kami et al. (2008)	3 (3F)	NA	36.3 ± 6.8 (31–44)	NA	Sucrose – tasteless	2	Uncorrected *p *<* *.001	SPM
Bender et al. (2009)	15 (6M, 9F)	R	25.4 ± NA (22–31)	NA	Taste (sucrose/ NaCl/ citric acid) – tasteless	5	FDR	SPM
Haase et al. (2009)	18 (9M, 9F)	NA	20.7 ± 1.0 (19–22)	12 h	Sucrose – tasteless; saccharin – tasteless; citric acid – tasteless; caffeine – tasteless; NaCl – tasteless; GMP – tasteless	116	Monte Carlo	AFNI
Veldhuizen et al. (2010)	15 (4M, 11F)	R	24 ± 4.9 (NA)	NA	High sucrose – low sucrose	19	FDR	SPM
Eldeghaidy et al. (2011)	13 (7M, 6F)	R	28 ± 8 (NA)	2 h	Sucrose – tasteless	13	Uncorrected *p *<* *.01 + k > 5	SPM
Nakamura et al. (2011)	20 (10M, 10F)	R	24.2 ± 2.7 (19–29)	3 h	(MSG + IMP) – tasteless; NaCl – tasteless	2	FWE	SPM
Cerf‐Ducastel et al. (2012)	18 (9M, 9F)	R	20.7 ± 1.0 (19–22)	12 h	Taste (sucrose/ saccharin/ NaCl/ citric acid/ caffeine/ GMP) – tasteless	25	Monte Carlo	AFNI
Green and Murphy (2012)	12 (5M, 7F)	NA	23.0 ± 2.3 (NA)	12 h	Saccharin – tasteless; sucrose – tasteless	37	Monte Carlo	AFNI, FSL
Nakamura et al. (2012)	20 (10M, 10F)	NA	24.2 ± 2.7 (19–29)	2 h	Sucrose – tasteless	1	FWE	SPM
Green et al. (2013)	24 (12M, 12F)	NA	36.3 ± 2.6 (19–54)	12 h	Sucrose – caffeine	7	Monte Carlo	AFNI
Avery et al. (2015)	20 (12M, 8F)	R	28 ± 7 (18–39)	NA	Sucrose – tasteless	7	Monte Carlo	AFNI
Total	238 (105M, 126F, 7 unidentified)					295		

a F, female. M, male.

b NA, not available in original paper. R, right‐handed.

c GMP, guanosine monophosphate. IMP, inosine monophosphate. MSG, monosodium glutamate.

d FDR, false discovery rate. FWE, family‐wise error. k, cluster size in units of contiguous voxels.

e FNI, Analysis of Functional NeuroImages. SPM, Statistical Parametric Mapping.

Five studies reported the body mass index (BMI; mean ± SD) of their participants (Haase et al., 2009, 23.7 without SD; Eldeghaidy et al., 2011, 24 ± 4; Green & Murphy, 2012, 25.0 ± 5.6; Green et al., 2013, 24 ± 2.7; Avery et al., 2015, 29 ± 6). All reported mean BMI values were below 30, the cut‐off threshold of obesity as defined by World Health Organization (World Health Organization, 2006). It should be noted that the mean BMI values from two studies (Avery et al., 2015; Green & Murphy, 2012) were within the range of overweight (BMI ≥ 25, World Health Organization, 2006). Moreover, Small et al. (2003) reported their participants were “of average weight and screened for obesity and malnutrition on the basis of their body mass index”. None of the studies reported the ethnic background of the participants.

Author affiliations revealed that five studies were from a San Diego (US) research group (Cerf‐Ducastel et al., 2012; Green & Murphy, 2012; Green et al., 2013; Haase et al., 2007, 2009), four from a New Haven/Chicago (US) team (Bender et al., 2009; Small et al., 2003; Veldhuizen et al., 2007, 2010), three from a Japanese team (Kami et al., 2008; Nakamura et al., 2011, 2012), two from an Oxford (UK) team (McCabe & Rolls, 2007; O'Doherty et al., 2001), one from a Nottingham (UK) team (Eldeghaidy et al., 2011), and one from an Oklahoma (US) team (Avery et al., 2015).

Among 16 studies (34 experiments), the effect of sweet taste was reported in 10 studies (14 experiments), salty taste reported in four studies (five experiments), umami taste reported in three studies (four experiments), bitter taste reported in two studies (three experiments), sour taste reported in one study (two experiments), and the combined effects reported in five studies (six experiments). Six studies reported results from multiple contrasts of basic taste stimuli (Green & Murphy, 2012; Haase et al., 2009; McCabe & Rolls, 2007; Nakamura et al., 2011; O'Doherty et al., 2001; Small et al., 2003). For the analyses, these results from different contrasts were treated as separate independent studies, which is a common and valid method to handle within‐subjects designs in ALE meta‐analyses using a modified ALE algorithm (Engelmann et al., 2012; Turkeltaub et al., 2002, 2012).

### 3.2 Overall ALE meta‐analysis results

**Figure 2:**
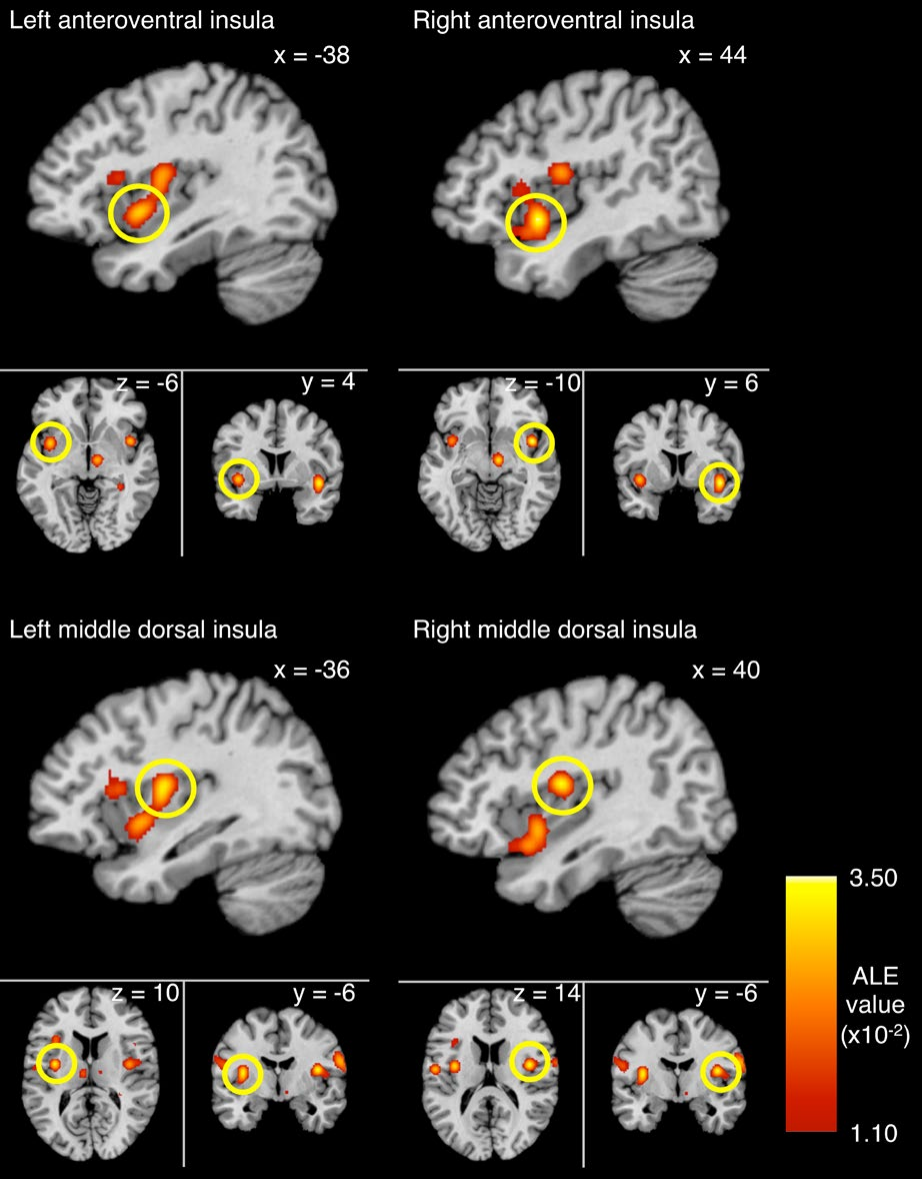
Localization of the significant activation likelihood estimation (ALE) in the bilateral insulae by taste stimulations overlaid onto a standard template (Colin27_T1_seg_MNI.nii) in Montreal Neurological Institute (MNI) space. Bilateral activation patterns were relatively symmetrical and focused on the anteroventral and middle dorsal parts. The map was generated using data from 238 individuals

The primary meta‐analysis pooled data across all 16 eligible studies. Results revealed nine statistically significant clusters activated by the effect of taste (Table 2). Four of these clusters involved the insula. Both anteroventral and middle dorsal parts of the bilateral insulae were involved (Figure 2). The other brain structures involved included the thalamus, pre‐/postcentral gyrus, hippocampus, and caudate. Sweet taste and taste in general contributed to every cluster reported, whereas bitter taste contributed to six, umami taste to five, salty taste to three, and sour taste to two.

**Table 2:** Locations of supra‐threshold clusters activated by taste stimulations as revealed by meta‐analysis

Cluster	Brain region^a^	Peak voxel MNI coordinates^b^			Cluster size (mm^3^)	ALE value (×10^−2^)	Contributing experiments				
							Total		Detailed breakdown		
		x	y	z			No.	%^c^	Taste	No.	%^b^
1	Anteroventral insula R	44	6	−10	3,464	3.53	10	29	Sweet	3	21
									Salty	1	20
									Umami	1	25
									Bitter	1	33
									General	4	67
2	Middle dorsal insula R	40	−6	14	2,008	3.46	8	24	Sweet	6	43
									Umami	1	25
									General	1	17
3	Middle dorsal insula L/	−36	−6	10	4,104	3.27	13	38	Sweet	6	43
	Anteroventral insula L	−*38*	*4*	−*6*		3.06			Salty	1	20
									Umami	1	25
									Bitter	1	33
									Sour	1	50
									General	3	50
4	Anterior insula L	−34	16	10	856	2.06	4	12	Sweet	3	21
									General	1	17
5	Thalamus R/	10	−14	−8	3,920	3.23	11	32	Sweet	6	43
	Mediodorsal thalamus L	−*6*	−*14*	*6*		2.25			Salty	1	20
									Bitter	1	33
									Sour	1	50
									General	2	33
6	Precentral gyrus R^b^	64	−4	22	2,400	3.05	9	26	Sweet	5	36
									Umami	2	50
									Bitter	1	33
									General	1	17
7	Postcentral gyrus L/	−54	−10	18	2,760	3.20	9	26	Sweet	7	50
	Precentral gyrus L	−*62*	−*2*	*24*		1.86			Umami	1	25
									General	1	17
8	Hippocampus R	32	−40	−2	1,104	2.31	5	15	Sweet	2	14
									Bitter	1	33
									General	2	33
9	Caudate R/	12	16	0	960	1.80	5	15	Sweet	3	21
	Caudate L	−*4*	*16*	*0*		1.46			Bitter	1	33
									General	1	17

Clusters were thresholded at *p *<* *.05 (cluster‐level family‐wise error corrected for multiple comparisons).

a L, left hemisphere. R, right hemisphere.

MNI, Montreal Neurological Institute. Italics indicate a peak fall under same cluster as preceding peak.

% calculated based on total experiment number (*n *= 34).

b % calculated based on experiment number of that particular taste (sweet = 14, salty = 5, umami = 4, bitter = 3, sour = 2, and general = 6).

c This cluster also covered the postcentral gyrus R.

Results of the supplementary meta‐analysis of ROI studies are described in Supplementary File 2.

### 3.3 MACM coactivation results

Results showed the VOIs located in the insula, pre‐/postcentral gyrus, and thalamus often coactivated with one another across all experiments indexed in the BrainMap database, whereas the caudate and hippocampus VOIs coactivated with a relatively limited number of brain regions only. In other words, the former group had a higher functional connectivity than the latter group. Brain structures that coactivated with at least 50% of the VOIs included the anterior insula, middle cingulate cortex, medial frontal gyrus, precentral gyrus, inferior parietal lobule, thalamus, and putamen (Table 3).

**Table 3:** Brain regions coactivated with each volume of interest (VOI) according to meta‐analytic connectivity modeling (MACM)

	VOI								
	AI	AVI and MI	AVI	MI	PoCG	PrCG	Tha	Cd	Hipp
	Left	Left	Right	Right	Left	Right	Bil	Bil	Right
AI									
Left	–	–	×		×	×	×	×	
Right	×	×	–		×	×	×	×	
MI									
Left		–	×	×	×	×	×		
Right		×		–	×	×	×		
MCC									
Bil	×	×	×	×	×	×	×	×	
MFG									
Bil	×	×	×	×	×	×	×		
PrCG									
Left	×	×	×	×	×	×	×		
Right	×	×		×	×	×	×		
PoCG									
Left		×	×	×	–	×	×		
Right		×		×	×	–	×		
IPL									
Left	×	×	×				×		
Right	×	×	×	×			×		
Precu									
Left							×		
Right	×								
Tha									
Left	×	×	×	×	×	×	–	×	
Right	×	×	×	×	×	×	–		
Amyg									
Left			×				×		
Right			×				×		
Puta									
Left	×	×	×		×	×	×	×	
Right	×	×	×		×	×	×	×	
Cd									
Left							×	–	
Right							×	–	
Culmen									
Left				×	×	×	×		
Right		×			×	×	×		
Hipp									
Right									×

Bil, bilateral; AI, anterior insula, Amyg, amygdala, AVI, anteroventral insula, Cd, caudate, Hipp, hippocampus, IPL, inferior parietal lobule, MCC, middle cingulate cortex, MFG, medial frontal gyrus, MI, middle insula, PoCG, postcentral gyrus, PrCG, precentral gyrus, Precu, precuneus, Puta, putamen, Tha, thalamus.

Each column represented the coactivation pattern of a VOI with other brain regions across selected experimental data stored in BrainMap database.

×, coactivation, –, not applicable, blank, no coactivation.

## 4 Discussion

Here, we used the most up‐to‐date ALE algorithm publicly available, and the newest recommended statistical thresholding technique, to perform an ALE meta‐analysis on fMRI data related to basic taste, and to identify patterns of connectivity related to such basic taste processing. We found bilateral activation in several areas, such as the thalamus, insula, and caudate, which was consistent across the eligible studies. The percentage of included experiments contributing to each of the significant clusters was in the range of 12–38%. This was comparable to results from van der Laan et al. (2011, 12–41%) and van Meer et al. (2015, 6–44% except two clusters at 75%). Various factors have been proposed as potential sources of the modest concurrence of studies, including variations in experimental designs, taste stimulations, MRI machines, analytical methodology, and participant characteristics (van der Laan & Smeets, 2015; van der Laan et al., 2011; van Meer et al., 2015). In addition, most of the significant clusters reported in this study were predominantly contributed by sweet taste, which would be expected given that most of the included studies used sweet taste stimulations.

### 4.1 Comparison of study inclusion with previous meta‐analytic studies

Nine of the primary studies from Veldhuizen et al. (2011) were excluded for being limited to ROI results only (Cerf‐Ducastel, Van de Moortele, MacLeod, Le Bihan, & Faurion, 2001; De Araujo, Rolls, Kringelbach, McGlone, & Phillips, 2003; O'Doherty, Deichmann, Critchley, & Dolan, 2002; Ogawa et al., 2005) or being PET studies (Kinomura et al., 1994; Small, Jones‐Gotman, Zatorre, Petrides, & Evans, 1997a; Small, Jones‐Gotman, Zatorre, Petrides, & Evans, 1997b; Zald, Hagen, & Pardo, 2002; Zald, Lee, Fluegel, & Pardo, 1998). Note that the Cerf‐Ducastel et al. (2001) study was referred to differently (Cerf‐Ducastel & Murphy, 2001) in the bibliography of the Veldhuizen et al. (2011) study. Similarly, 10 studies from Kurth et al. (2010) were excluded for (1) having no results attributed specifically to basic taste stimulation (Berns, McClure, Pagnoni, & Montague, 2001; De Araujo & Rolls, 2004; Kobayashi et al., 2004); (2) reporting on ROI results only (De Araujo, Rolls, et al., 2003; Ogawa et al., 2005; Schoenfeld et al., 2004); (3) having no results reported in Talairach or MNI coordinates (De Araujo, Kringelbach, Rolls, & Hobden, 2003; Schoenfeld et al., 2004); (4) not identifying the analytic software used (Francis et al., 1999); and (5) being PET studies (Zald et al., 1998, 2002). Finally, the total number of studies (*n *= 16) included in the current meta‐analysis was slightly larger than those two studies.

### 4.2 Comparison of activated regions with previous meta‐analytic studies

We found that a number of taste‐activated regions were consistent with the results of previous meta‐analysis. These included the mediodorsal thalamus, anteroventral and middle dorsal insula, and postcentral gyrus. In addition, we found significant clusters activated by basic taste stimulations in the hippocampus and caudate that was not reported from Veldhuizen et al. (2011). However, our study did not support the previous findings of significant clusters in the orbitofrontal cortex and anterior cingulate gyrus activated by basic taste stimulations (Veldhuizen et al., 2011). To verify this difference, we performed an exploratory analysis on the pooled data using more lenient statistical thresholds. With a threshold of *p *<* *.05 with voxel‐wise FDR correction, we observed additional activation in right orbitofrontal cortex (peak voxel: 42, 38, −16; ALE value: 1.76 × 10^−2^; cluster volume: 264 mm^3^). With a threshold of uncorrected *p *<* *.001, we observed activation in anterior cingulate cortex (peak voxel: 16, 46, −10; ALE value: 1.41 × 10^−2^; cluster volume: 168 mm^3^). These observations could be accounted for by the differences in the studies included in the meta‐analyses. In the current meta‐analysis, orbitofrontal cortex and anterior cingulate gyrus were reported in seven and 10 of the included studies, respectively. However, the reported coordinates varied across the studies and were not consistent. This might partially explain why they were not detected under more stringent statistical thresholds.

### 4.3 Reported roles of activated regions from previous neuroimaging studies

Previous studies have suggested that different parts of the insula are responsible for processing different aspects of taste perception. For instance, Small et al. (2003) reported that the activation in anterior insula was more related to the valence aspect of taste (i.e., whether a taste is pleasant or aversive); whereas the middle insula was more related to processing taste intensity. The significant clusters in anterior insula reported in this study were close to the clusters previously reported for valence involvement (Dalenberg, Hoogeveen, Renken, Langers, & ter Horst, 2015; Jabbi, Swart, & Keysers, 2007; Small et al., 2003). Similarly, the significant clusters in middle insula reported in this study were close to the ones previously reported for processing intensity/ concentration (Kobayakawa, Saito, Gotow, & Ogawa, 2008; Small et al., 2003; Spetter, Smeets, de Graaf, & Viergever, 2010).

It is known that the thalamus is a gateway through which peripheral neural signals pass through to reach the cortex. For taste processing, the thalamus was activated by detecting the presence of taste (Haase et al., 2007; Yeung, Tanabe, Suen, & Goto, 2016), differences in state of satiety (e.g., hunger vs. satiety) of participants (Haase et al., 2009), and processing valence (Cerf‐Ducastel et al., 2012). On the other hand, the precentral and postcentral gyri were involved in taste detection (Kobayashi et al., 2004) and processing valence (Berns et al., 2001; Calder et al., 2007).

Numerous taste‐relevant conditions activated the insula, thalamus, and pre‐/postcentral gyrus, and some of the conditions activated them in groups. This was consistent with the MACM coactivation results that showed frequent coactivation (functional connectivity) among these activated clusters. From MACM results, these taste‐relevant VOIs also often coactivated with the middle cingulate cortex, medial frontal gyrus, inferior parietal lobule, and putamen. These regions appear to integrate taste sensation with other perceptual contexts, such as attentiveness (Lawrence, Ross, Hoffmann, Garavan, & Stein, 2003), taste–smell interactions (Seo et al., 2013) and the emotional aspect of chemosensory perception (Wicker et al., 2003).

The remaining activated regions from this meta‐analysis were the hippocampus and caudate. The hippocampus can be activated by taste stimulations (Gautier et al., 1999; Haase et al., 2007), and by recall of taste stimuli (Haase et al., 2009). The caudate, on the other hand, was responsible for processing the pleasantness and reward value of taste stimuli (Cerf‐Ducastel et al., 2012; Green & Murphy, 2012).

Earlier studies have proposed a degree of laterality in cortical taste processing. The inferior (i.e., ventral) insula appeared to be preferably activated on the contralateral side of the dominant hand (Faurion et al., 1999). In addition, right hemisphere dominance was previously found for taste‐related insula activation (Small et al., 2003). However, both our results and those of Veldhuizen et al. (2011) demonstrate a relatively balanced map of taste‐related activations. Bilateral activation is not necessarily contradictory to lateralization, as lateralized activity can represent specific aspects of taste processing, such as intensity and pleasantness (Dalenberg et al., 2015). Future studies with a larger sample size will be needed to better describe taste‐related lateralization, as well as to determine the possible relationship of handedness in cortical taste processing.

### 4.4 Contribution of each taste to the clusters reported in this meta‐analysis

We noted that every activated cluster reported in this study was the result of contributions by multiple tastes. Notably, sweet taste and taste in general contributed to all clusters, whereas sour taste contributed to two clusters at the thalamus and left insula only. As mentioned previously, none of the individual basic tastes had enough experiments available (*n *= 17) for a proper independent meta‐analysis (Eickhoff et al., 2016). Though the current results might suggest that each taste contributed to the activated clusters in different ratios, the differences across tastes revealed from the current results could be largely due to the unbalanced employment of tastes in the included studies. Moreover, past studies did not report on particular anatomical structures in the brain that were consistently activated by specific taste(s) only.

### 4.5 Limitations and future prospects

One limitation of this study was the relatively small number of studies eligible after screening with stringent criteria. However, the size of our final dataset was comparable to that of Veldhuizen et al. (2011), as well as other meta‐analyses with similar topics such as swallowing (Sörös, Inamoto, & Martin, 2009), smoking (Engelmann et al., 2012), and viewing food cues (van der Laan et al., 2011). In addition, our total of 34 experiments was double of the recommended minimum of 17 (Eickhoff et al., 2016) needed to control for excessive contributions by specific experiments. One further limitation was the potential for confounding factors related to swallowing of the taste liquids, which in itself activates various brain areas such as the right insula and hippocampus, bilateral pre‐/postcentral gyrus, and left thalamus (Little et al., 2014; Sörös et al., 2009; Spetter, de Graaf, Mars, Viergever, & Smeets, 2014). Most taste processing study protocols required ingestion of very small amounts of flavored liquids. However, the period of swallowing can be modeled out from the baseline during analysis. In addition, there were delivery systems designed to eliminate such need to swallow (Goto, Yeung, Suen, Fong, & Ninomiya, 2015; Kami et al., 2008). Thus, we believe swallowing‐related effects on the data to be minimal.

We identified three suggestions for future studies in the field. First, we noted that the five basic tastes were not yet studied in a balanced way; for example, there was only one study of pure sour taste and two of pure bitter taste in the final inclusion list of this report. Therefore, more studies on sour taste (as well as the others) are needed to reliably map the cortical representations of the individual tastes. Second, we believe future studies should provide BMI data on the study groups, which we found to go mostly unreported in our dataset (only five out of 16 included studies reported BMI). This is needed as one notable study found that people with obesity (*n *= 12) had larger brain responses to sweet and bitter tastes than normal‐weight (*n *= 12) people (Szalay et al., 2012). It is still unclear if such an increase in activation exists among overweight people. Finally, we feel future studies should report the effect sizes, to allow for effect‐size‐based meta‐analyses that could benefit the overall analysis of the relationship between brain activation and taste stimulation.

## 5 Conclusion

In conclusion, our results indicated that previous fMRI studies have consistently identified a pattern of activity related to basic taste stimulation including the bilateral anterior and middle insula, thalamus, caudate, pre‐/postcentral gyrus, and right hippocampus. Connectivity analysis suggests that the above results represent a core network of taste processing, which is functionally connected to a wider network relevant to integrating taste processing with other perceptual contexts, and includes the middle cingulate cortex, medial frontal gyrus, inferior parietal lobule, and putamen. Taken together, our meta‐analysis validates and confirms previous results (Veldhuizen et al., 2011), complements those data by providing MNI‐based coordinates for activated areas, indicates that sweet taste was the predominant contributor to the activation results, and provides novel information on the functional connectivity necessary for basic taste sensation and its cognitive processing.

## Conflict of Interest

The authors declare no competing financial interests.
