# Supplementary material for: Basic taste processing recruits bilateral anteroventral and middle dorsal insulae: An activation likelihood estimation meta‐analysis of fMRI studies
Source: Brain Behav. 2017 Mar 10;7(4):e00655. doi: 10.1002/brb3.655 (PMC5390838; doi:10.1002/brb3.655)
Supplement: Supplementary file 1 [file BRB3-7-e00655-s001.docx]

**Supplementary File 1.**

The full list of 85 excluded publications from main meta-analysis (in alphabetical order).

Berns, G.S., McClure, S.M., Pagnoni, G., Montague, P.R. 2001. Predictability modulates human brain response to reward. J Neurosci. 21, 2793-2798.

Bohon, C. 2014. Greater emotional eating scores associated with reduced frontolimbic activation to palatable taste in adolescents. Obesity 22, 1814-1820.

Cerf, B., Lebihan, D., Moortele, P., Mac Leod, P., Faurion, A. 1998. Functional Lateralization of Human Gustatory Cortex Related to Handedness Disclosed by fMRI Study. Ann. N. Y. Acad. Sci. 855, 575-578.

Cerf-Ducastel, B., Murphy, C. 2004. Validation of a stimulation protocol suited to the investigation of odor–taste interactions with fMRI. Physiol. Behav. 81, 389-396.

Cerf-Ducastel, B., Van de Moortele, P.-F., MacLeod, P., Le Bihan, D., Faurion, A. 2001. Interaction of gustatory and lingual somatosensory perceptions at the cortical level in the human: a functional magnetic resonance imaging study. Chem. Senses 26, 371-383.

Connolly, L., Coveleskie, K., Kilpatrick, L., Labus, J., Ebrat, B., Stains, J., Jiang, Z., Tillisch, K., Raybould, H., Mayer, E. 2013. Differences in brain responses between lean and obese women to a sweetened drink. Neurogastroenterol. Motil. 25, 579-e460.

Cornier, M.-A., Shott, M.E., Thomas, E.A., Bechtell, J.L., Bessesen, D.H., Tregellas, J.R., Frank, G.K. 2015. The effects of energy balance, obesity-proneness and sex on the neuronal response to sweet taste. Behav. Brain Res. 278, 446-452.

Cowdrey, F.A., Park, R.J., Harmer, C.J., McCabe, C. 2011. Increased neural processing of rewarding and aversive food stimuli in recovered anorexia nervosa. Biol. Psychiatry 70, 736-743.

Dalenberg, J.R., Hoogeveen, H.R., Renken, R.J., Langers, D.R., ter Horst, G.J. 2015. Functional specialization of the male insula during taste perception. NeuroImage 119, 210-220.

De Araujo, I., Kringelbach, M., Rolls, E., Hobden, P. 2003a. Representation of umami taste in the human brain. J. Neurophysiol. 90, 313-319.

De Araujo, I.E., Kringelbach, M.L., Rolls, E.T., McGlone, F.,2003b. Human cortical responses to water in the mouth, and the effects of thirst. J. Neurophysiol. 90, 1865-1876.

De Araujo, I.E., Lin, T., Veldhuizen, M.G., Small, D.M. 2013. Metabolic regulation of brain response to food cues. Curr. Biol. 23, 878-883.

De Araujo, I.E., Rolls, E.T. 2004. Representation in the human brain of food texture and oral fat. J. Neurosci. 24, 3086-3093.

De Araujo, I.E., Rolls, E.T., Kringelbach, M.L., McGlone, F., Phillips, N. 2003c. Taste‐olfactory convergence, and the representation of the pleasantness of flavour, in the human brain. Eur. J. Neurosci. 18, 2059-2068.

Di Salle, F., Cantone, E., Savarese, M.F., Aragri, A., Prinster, A., Nicolai, E., Sarnelli, G., Iengo, M., Buyckx, M., Cuomo, R. 2013. Effect of carbonation on brain processing of sweet stimuli in humans. Gastroenterol. 145, 537-539.

Ebisch, S.J., Bello, A., Spitoni, G.F., Perrucci, M.G., Gallese, V., Committeri, G., Pastorelli, C., Pizzamiglio, L. 2015. Emotional susceptibility trait modulates insula responses and functional connectivity in flavor processing. Front. Behav. Neurosci. 9.

Faurion, A., Cerf, B., Le Bihan, D., Pilliasa, A.M. 1998. fMRI study of taste cortical areas in humans. Ann. N. Y. Acad. Sci. 855, 535-545.

Faurion, A., Cerf, B., Van De Moortele, P.-F., Lobel, E., Mac Leod, P., Le Bihan, D. 1999. Human taste cortical areas studied with functional magnetic resonance imaging: evidence of functional lateralization related to handedness. Neurosci. Lett. 277, 189-192.

Filbey, F.M., Myers, U.S., DeWitt, S. 2012. Reward circuit function in high BMI individuals with compulsive overeating: similarities with addiction. NeuroImage 63, 1800-1806.

Francis, S., Rolls, E.T., Bowtell, R., McGlone, F., O'Doherty, J., Browning, A., Clare, S., Smith, E. 1999. The representation of pleasant touch in the brain and its relationship with taste and olfactory areas. Neuroreport 10, 453-459.

Frank, G.K., Collier, S., Shott, M.E., O’Reilly, R.C. 2016. Prediction error and somatosensory insula activation in women recovered from anorexia nervosa. J. Psychiatry Neurosci.1, 8872147.

Frank, G.K., Kaye, W.H., Carter, C.S., Brooks, S., May, C., Fissell, K., Stenger, V.A. 2003. The evaluation of brain activity in response to taste stimuli—a pilot study and method for central taste activation as assessed by event-related fMRI. J. Neurosci. Methods 131, 99-105.

Frank, G.K., Reynolds, J.R., Shott, M.E., Jappe, L., Yang, T.T., Tregellas, J.R., O'Reilly, R.C. 2012. Anorexia nervosa and obesity are associated with opposite brain reward response. Neuropsychopharmacol. 37, 2031-2046.

Frank, G.K., Reynolds, J.R., Shott, M.E., O'Reilly, R.C. 2011. Altered temporal difference learning in bulimia nervosa. Biol. Psychiatry 70, 728-735.

Gagnon, L., Kupers, R., Ptito, M. 2015. Neural correlates of taste perception in congenital blindness. Neuropsychologia 70, 227-234.

Gagnon, L., Vestergaard, M., Madsen, K., Karstensen, H.G., Siebner, H., Tommerup, N., Kupers, R., Ptito, M. 2014. Neural correlates of taste perception in congenital olfactory impairment. Neuropsychologia 62, 297-305.

Ge, T., Feng, J., Grabenhorst, F., Rolls, E.T. 2012. Componential Granger causality, and its application to identifying the source and mechanisms of the top–down biased activation that controls attention to affective vs sensory processing. NeuroImage 59, 1846-1858.

Grabenhorst, F., Rolls, E.T. 2008. Selective attention to affective value alters how the brain processes taste stimuli. Eur. J. Neurosci. 27, 723-729.

Grabenhorst, F., Rolls, E.T., Bilderbeck, A. 2008. How cognition modulates affective responses to taste and flavor: top-down influences on the orbitofrontal and pregenual cingulate cortices. Cereb. Cortex 18, 1549-1559.

Green, E., Jacobson, A., Haase, L., Murphy, C. 2011. Reduced nucleus accumbens and caudate nucleus activation to a pleasant taste is associated with obesity in older adults. Brain Res. 1386, 109-117.

Green, E., Jacobson, A., Haase, L., Murphy, C. 2015. Neural correlates of taste and pleasantness evaluation in the metabolic syndrome. Brain Res. 1620, 57-71.

Guest, S., Grabenhorst, F., Essick, G., Chen, Y., Young, M., McGlone, F., de Araujo, I., Rolls, E.T. 2007. Human cortical representation of oral temperature. Physiol. Behav. 92, 975-984.

Hoogeveen, H.R., Dalenberg, J.R., Renken, R.J., ter Horst, G.J., Lorist, M.M. 2015. Neural processing of basic tastes in healthy young and older adults—an fMRI study. NeuroImage 119, 1-12.

Hort, J., Ford, R.A., Eldeghaidy, S., Francis, S.T. 2016. Thermal taster status: Evidence of cross‐modal integration. Hum. Brain Mapp. 37, 2263-2275.

Hummel, C., Frasnelli, J., Gerber, J., Hummel, T. 2007. Cerebral processing of gustatory stimuli in patients with taste loss. Behav. Brain Res. 185, 59-64.

Iannilli, E., Singh, P.B., Schuster, B., Gerber, J., Hummel, T. 2012. Taste laterality studied by means of umami and salt stimuli: an fMRI study. NeuroImage 60, 426-435.

Jabbi, M., Swart, M., Keysers, C. 2007. Empathy for positive and negative emotions in the gustatory cortex. NeuroImage 34, 1744-1753.

Jacobson, A., Green, E., Murphy, C. 2010. Age-related functional changes in gustatory and reward processing regions: An fMRI study. NeuroImage 53, 602-610.

James, G.A., Li, X., DuBois, G.E., Zhou, L., Hu, X.P. 2009. Prolonged insula activation during perception of aftertaste. Neuroreport 20, 245-250.

Kareken, D.A., Dzemidzic, M., Oberlin, B.G., Eiler, W.J. 2013. A preliminary study of the human brain response to oral sucrose and its association with recent drinking. Alcohol. Clin. Exp. Res. 37, 2058-2065.

Kawakami, S., Sato, H., Sasaki, A.T., Tanabe, H.C., Yoshida, Y., Saito, M., Toyoda, H., Sadato, N., Kang, Y. 2015. The brain mechanisms underlying the perception of pungent taste of capsaicin and the subsequent autonomic responses. Front. Hum. Neurosci. 9, 720.

Kerr, K.L., Avery, J.A., Barcalow, J.C., Moseman, S.E., Bodurka, J., Bellgowan, P.S., Simmons, W.K. 2015. Trait impulsivity is related to ventral ACC and amygdala activity during primary reward anticipation. Soc. Cogn. Affect. Neurosci. 10, 36-42.

King, A.B., Menon, R.S., Hachinski, V., Cechetto, D.F. 1999. Human forebrain activation by visceral stimuli. J. Comp. Neurol. 413, 572-582.

Kobayashi, M., Takeda, M., Hattori, N., Fukunaga, M., Sasabe, T., Inoue, N., Nagai, Y., Sawada, T., Sadato, N., Watanabe, Y. 2004. Functional imaging of gustatory perception and imagery:“top-down” processing of gustatory signals. NeuroImage 23, 1271-1282.

Luo, Q., Ge, T., Grabenhorst, F., Feng, J., Rolls, E.T. 2013. Attention-dependent modulation of cortical taste circuits revealed by granger causality with signal-dependent noise. PLoS Comput. Biol. 9, e1003265.

Luo, S., Monterosso, J.R., Sarpelleh, K., Page, K.A. 2015. Differential effects of fructose versus glucose on brain and appetitive responses to food cues and decisions for food rewards. Proc. Natl. Acad. Sci. 112, 6509-6514.

Marciani, L., Pfeiffer, J.C., Hort, J., Head, K., Bush, D., Taylor, A.J., Spiller, R.C., Francis, S., Gowland, P.A. 2006. Improved methods for fMRI studies of combined taste and aroma stimuli. J. Neurosci. Methods 158, 186-194.

Mascioli, G., Berlucchi, G., Pierpaoli, C., Salvolini, U., Barbaresi, P., Fabri, M., Polonara, G. 2015. Functional MRI cortical activations from unilateral tactile-taste stimulations of the tongue. Physiol. Behav. 151, 221-229.

McCabe, C., Woffindale, C., Harmer, C.J., Cowen, P.J. 2012. Neural processing of reward and punishment in young people at increased familial risk of depression. Biol. Psychiatry 72, 588-594.

Nakamura, Y., Tokumori, K., Tanabe, H.C., Yoshiura, T., Kobayashi, K., Nakamura, Y., Honda, H., Yoshiura, K., Goto, T.K. 2013. Localization of the primary taste cortex by contrasting passive and attentive conditions. Exp. Brain Res. 227, 185-197.

Nitschke, J.B., Dixon, G.E., Sarinopoulos, I., Short, S.J., Cohen, J.D., Smith, E.E., Kosslyn, S.M., Rose, R.M., Davidson, R.J. 2006. Altering expectancy dampens neural response to aversive taste in primary taste cortex. Nature Neurosci. 9, 435-442.

O'Doherty, J.P., Dayan, P., Friston, K., Critchley, H., Dolan, R.J. 2003. Temporal difference models and reward-related learning in the human brain. Neuron 38, 329-337.

O'Doherty, J.P., Deichmann, R., Critchley, H.D., Dolan, R.J. 2002. Neural responses during anticipation of a primary taste reward. Neuron 33, 815-826.

Ogawa, H., Wakita, M., Hasegawa, K., Kobayakawa, T., Sakai, N., Hirai, T., Yamashita, Y., Saito, S. 2005. Functional MRI detection of activation in the primary gustatory cortices in humans. Chem. Senses 30, 583-592.

Pauli, W.M., Larsen, T., Collette, S., Tyszka, J.M., Seymour, B., O'Doherty, J.P. 2015. Distinct Contributions of Ventromedial and Dorsolateral Subregions of the Human Substantia Nigra to Appetitive and Aversive Learning. J. Neurosci. 35, 14220-14233.

Rolls, E.T., Kellerhals, M.B., Nichols, T.E. 2015. Age differences in the brain mechanisms of good taste. NeuroImage 113, 298-309.

Rudenga, K., Green, B., Nachtigal, D., Small, D. 2010. Evidence for an integrated oral sensory module in the human anterior ventral insula. Chem. Senses 35, 693-703.

Rudenga, K., Small, D. 2012. Amygdala response to sucrose consumption is inversely related to artificial sweetener use. Appetite 58, 504-507.

Rudenga, K.J., Small, D.M. 2013. Ventromedial prefrontal cortex response to concentrated sucrose reflects liking rather than sweet quality coding. Chem. Senses, 38, 585-594.

Sarinopoulos, I., Dixon, G.E., Short, S.J., Davidson, R.J., Nitschke, J.B. 2006. Brain mechanisms of expectation associated with insula and amygdala response to aversive taste: implications for placebo. Brain Behav. Immun. 20, 120-132.

Schoenfeld, M., Neuer, G., Tempelmann, C., Schüßler, K., Noesselt, T., Hopf, J.-M., Heinze, H.-J. 2004. Functional magnetic resonance tomography correlates of taste perception in the human primary taste cortex. Neurosci.127, 347-353.

Serra‐Grabulosa, J.M., Adan, A., Falcón, C., Bargalló, N. 2010. Glucose and caffeine effects on sustained attention: an exploratory fMRI study. Hum. Psychopharmacol. 25, 543-552.

Seubert, J., Ohla, K., Yokomukai, Y., Kellermann, T., Lundström, J.N. 2015. Superadditive opercular activation to food flavor is mediated by enhanced temporal and limbic coupling. Hum. Brain Mapp. 36, 1662-1676.

Shott, M., Cornier, M., Mittal, V., Pryor, T., Orr, J., Brown, M., Frank, G. 2015. Orbitofrontal cortex volume and brain reward response in obesity. Int. J. Obes. 39, 214-221.

Singh, P.B., Hummel, T., Gerber, J.C., Landis, B.N., Iannilli, E. 2015. Cerebral processing of umami: A pilot study on the effects of familiarity. Brain Res.1614, 67-74.

Small, D.M., Voss, J., Mak, Y.E., Simmons, K.B., Parrish, T., Gitelman, D. 2004. Experience-dependent neural integration of taste and smell in the human brain. J. Neurophysiol. 92, 1892-1903.

Smeets, P.A., de Graaf, C., Stafleu, A., van Osch, M.J., van der Grond, J. 2005. Functional magnetic resonance imaging of human hypothalamic responses to sweet taste and calories. Am. J. Clin. Nutr. 82, 1011-1016.

Smits, M., Peeters, R.R., Van Hecke, P., Sunaert, S. 2007. A 3 T event-related functional magnetic resonance imaging (fMRI) study of primary and secondary gustatory cortex localization using natural tastants. Neuroradiol. 49, 61-71.

Spetter, M., Smeets, P., De Graaf, C., Viergever, M. 2010. Representation of sweet and salty taste intensity in the brain. Chem. Senses 35, 831-840.

Szalay, C., Aradi, M., Schwarcz, A., Orsi, G., Perlaki, G., Németh, L., Hanna, S., Takács, G., Szabó, I., Bajnok, L. 2012. Gustatory perception alterations in obesity: an fMRI study. Brain Res. 1473, 131-140.

Thomas, J.M., Higgs, S., Dourish, C.T., Hansen, P.C., Harmer, C.J., McCabe, C. 2015. Satiation attenuates BOLD activity in brain regions involved in reward and increases activity in dorsolateral prefrontal cortex: an fMRI study in healthy volunteers. Am. J. Clin. Nutr. 101, 697-704.

Topolovec, J.C., Gati, J.S., Menon, R.S., Shoemaker, J.K., Cechetto, D.F. 2004. Human cardiovascular and gustatory brainstem sites observed by functional magnetic resonance imaging. J. Comp. Neurol. 471, 446-461.

Uher, R., Treasure, J., Heining, M., Brammer, M.J., Campbell, I.C. 2006. Cerebral processing of food-related stimuli: effects of fasting and gender. Behav. Brain Res. 169, 111-119.

van Rijn, I., de Graaf, C., Smeets, P.A. 2015. Tasting calories differentially affects brain activation during hunger and satiety. Behav. Brain Res. 279, 139-147.

Veldhuizen, M.G., Douglas, D., Aschenbrenner, K., Gitelman, D.R., Small, D.M. 2011. The anterior insular cortex represents breaches of taste identity expectation. J. Neurosci.31, 14735-14744.

Veldhuizen, M.G., Gitelman, D.R., Small, D.M. 2012. An fMRI study of the interactions between the attention and the gustatory networks. Chemosens. Percept. 5, 117-127.

Veldhuizen, M.G., Small, D.M. 2011. Modality-specific neural effects of selective attention to taste and odor. Chem. Senses 36, 747-760.

Vidarsdottir, S., Smeets, P.A., Eichelsheim, D.L., van Osch, M.J., Viergever, M.A., Romijn, J.A., van der Grond, J., Pijl, H. 2007. Glucose ingestion fails to inhibit hypothalamic neuronal activity in patients with type 2 diabetes. Diabetes 56, 2547-2550.

Wagner, A., Aizenstein, H., Frank, G.K., Figurski, J., May, J.C., Putnam, K., Fischer, L., Bailer, U.F., Henry, S.E., McConaha, C. 2006. Neural correlates of habituation to taste stimuli in healthy women. Psychiatry Res. Neuroimaging 147, 57-67.

Wagner, A., Aizenstein, H., Mazurkewicz, L., Fudge, J., Frank, G.K., Putnam, K., Bailer, U.F., Fischer, L., Kaye, W.H. 2008. Altered insula response to taste stimuli in individuals recovered from restricting-type anorexia nervosa. Neuropsychopharmacol 33, 513-523.

Wagner, A., Simmons, A.N., Oberndorfer, T.A., Frank, G.K., McCurdy-McKinnon, D., Fudge, J.L., Yang, T.T., Paulus, M.P., Kaye, W.H. 2015. Altered sensitization patterns to sweet food stimuli in patients recovered from anorexia and bulimia nervosa. Psychiatry Res. Neuroimaging 234, 305-313.

Wang, J.-L., Yang, Q., Hajnal, A., Rogers, A.M. 2015. A pilot functional MRI study in Roux-en-Y gastric bypass patients to study alteration in taste functions after surgery. Surg. Endosc. 30, 892-898.

Woods, A.T., Lloyd, D.M., Kuenzel, J., Poliakoff, E., Dijksterhuis, G.B., Thomas, A. 2011. Expected taste intensity affects response to sweet drinks in primary taste cortex. Neuroreport 22, 365-369.

Yan, X., Zhang, J., Gong, Q., Weng, X. 2011. Appetite at high altitude: an fMRI study on the impact of prolonged high-altitude residence on gustatory neural processing. Exp. Brain Res. 209, 495-499.

Yeung, A.W.K., Tanabe, H.C., Suen, J.L.K., Goto, T.K. 2016. Taste intensity modulates effective connectivity from the insular cortex to the thalamus in humans. NeuroImage 135, 214-222.
