# Supplementary material for: Basic taste processing recruits bilateral anteroventral and middle dorsal insulae: An activation likelihood estimation meta‐analysis of fMRI studies
Source: Brain Behav. 2017 Mar 10;7(4):e00655. doi: 10.1002/brb3.655 (PMC5390838; doi:10.1002/brb3.655)
Supplement: Supplementary file 2 [file BRB3-7-e00655-s002.docx]

**Supplementary File 2.**

**1. Introduction**

This file reports the supplementary meta-analysis of 14 studies reporting on region-of-interest (ROI) results only. This was to supplement the main meta-analysis reporting on whole brain results.

**2. Materials and Methods**

For details of literature search and selection criteria, please refer to the main text. The ROI studies included in this supplementary analysis fulfilled the other four selection criteria, namely: reporting results from systemic disease-free participants, results with brain activations attributable to basic taste stimulations, results in Talairach or Montreal Neurological Institute (MNI) spaces and reporting software used for analysis.

The procedures for conducting the meta-analysis with activation likelihood estimation (ALE) and result visualization were the same as for the main meta-analysis reported in the main text. For this list of ROI studies, however, we did not perform meta-analytic connectivity modeling (MACM) as it was more relevant for whole brain results.

**3. Results**

3.1 Study and participant profiles

The data from 14 ROI studies included in the supplementary meta-analysis involved 113 clusters of coordinates from 22 experiments utilizing 242 participants (96 males, 141 females, 5 unidentified) (Table S1). Each study enrolled 5–34 participants. Participants in eight studies were predominantly right-handed, while six studies did not report handedness. Participants were mainly 20–40 years old. Their fasting time before brain scanning ranged from 2–3 h. All studies used SPM for processing data. Participants in three studies (Ogawa et al., 2005; Nakamura et al., 2013; Yeung et al., 2016) did not need to swallow the taste sample liquids, while those in the remaining 11 studies needed to do so.

Five studies reported the body mass index (BMI; mean ± SD) of their participants (Veldhuizen et al., 2011, 24.9 ± 5.7; Rudenga et al., 2012, 23.4 ± 2.6; Rudenga et al., 2013, 23.4 ± 2.6; vin Rijn et al., 2015, 22.6 ± 1.4; Yeung et al., 2016, 20.2 ± 2.1). All reported mean BMI values were below 25, the cut-off threshold of overweight as defined by World Health Organization (World Health Organization, 2006).

Author affiliations revealed that four from an Oxford (UK) team (O’Doherty et al., 2002; de Araujo et al., 2003; Guest et al., 2007; Grabenhorst et al., 2008), three from a New Haven/Chicago (US) team (Veldhuizen et al., 2011; Rudenga et al., 2012, 2013), two from a Hong Kong/Japan team (Nakamura et al., 2013; Yeung et al., 2016), one study were from a San Diego (US) research group (Cerf-Ducastel et al., 2001), one from a Kumamoto (Japan) team (Ogawa et al., 2005), one from a Wageningen/Groningen (Netherlands) team (Dalenberg et al., 2015), one from a Wageningen/Utrecht (Netherlands) team (vin Rijn et al., 2015) and one from a German team (Singh et al., 2015).

Among 14 studies (22 experiments), the effect of sweet taste was reported in seven studies (seven experiments), salty taste reported in four studies (seven experiments), umami taste reported in two studies (five experiments), the general effect of taste reported in three studies (three experiments), but bitter and sour tastes were reported in none of the studies. Four studies reported results from multiple contrasts of basic taste stimuli (O’Doherty et al., 2002; Grabenhorst et al., 2008; Singh et al., 2015; Yeung et al., 2016). For the analyses, these results from different contrasts were treated as separate independent studies, which is a common and valid method to handle within-subjects designs in ALE meta-analyses using a modified ALE algorithm (Turkeltaub et al., 2002, 2012; Engelmann et al., 2012).

3.2 Overall ALE meta-analysis results

The primary meta-analysis pooled data across all fourteen eligible studies. Results revealed five statistically significant clusters activated by the effect of taste (Table S2). Three of these five clusters involved the insula. Both anterior and middle parts of the right insula were involved, while only the middle part of the left insula was involved (Fig. S1). The other brain structures involved included the thalamus and pre-/post-central gyrus, both of which were activated on the right hemisphere. Sweet and salty tastes contributed to every cluster reported, whereas general effect of taste contributed to four clusters except the one in left middle insula. Umami taste contributed to all three clusters in the insula but not the others. As bitter and sour tastes were not involved in the included studies of this supplementary meta-analysis, their contributions to these clusters could not be determined.

**4. Discussion**

Comparing to the main meta-analysis with whole brain data from 238 participants in 34 experiments, this supplementary meta-analysis involved ROI-limited data from similar amount of participants in fewer experiments (22 as compared to 34). Hence, it was expected to observe fewer significant clusters in similar locations resulted in this analysis (five as compared to nine). In terms of locations of peak voxels, cluster B (Table S2) was close to cluster 2 (Table 2 of main text); cluster C was close to cluster 3; and cluster D was close to cluster 5. Besides, cluster A was slightly more dorsal to cluster 1, while cluster E was slightly more posterior to cluster 7. Similar to main meta-analysis, few studies included in the supplementary meta-analysis reported participants’ BMI. Besides, as we reported that data from bitter and sour tastes were very limited from the included studies in the main meta-analysis, they were totally absent in the 14 studies included in this supplementary analysis.

**References**

Cerf-Ducastel, B., Van de Moortele, P.-F., MacLeod, P., Le Bihan, D., Faurion, A. 2001. Interaction of gustatory and lingual somatosensory perceptions at the cortical level in the human: a functional magnetic resonance imaging study. Chem. Senses 26, 371-383.

Dalenberg, J.R., Hoogeveen, H.R., Renken, R.J., Langers, D.R., ter Horst, G.J. 2015. Functional specialization of the male insula during taste perception. Neuroimage 119, 210-220.

De Araujo, I.E., Rolls, E.T., Kringelbach, M.L., McGlone, F., Phillips, N. 2003b. Taste-olfactory convergence, and the representation of the pleasantness of flavour, in the human brain. Eur. J. Neurosci. 18, 2059-2068.

Engelmann, J.M., Versace, F., Robinson, J.D., Minnix, J.A., Lam, C.Y., Cui, Y., et al. 2012. Neural substrates of smoking cue reactivity: a meta-analysis of fMRI studies. Neuroimage 60, 252-262.

Grabenhorst, F., Rolls, E.T., Bilderbeck, A. 2008. How cognition modulates affective responses to taste and flavor: top-down influences on the orbitofrontal and pregenual cingulate cortices. Cereb. Cortex 18, 1549-1559.

Guest, S., Grabenhorst, F., Essick, G., Chen, Y., Young, M., McGlone, F., et al. 2007. Human cortical representation of oral temperature. Physiol. Behav. 92, 975-984.

Nakamura, Y., Tokumori, K., Tanabe, H.C., Yoshiura, T., Kobayashi, K., Nakamura, Y., et al. 2013. Localization of the primary taste cortex by contrasting passive and attentive conditions. Exp. Brain Res. 227, 185-197.

O'Doherty, J.P., Deichmann, R., Critchley, H.D., Dolan, R.J. 2002. Neural responses during anticipation of a primary taste reward. Neuron 33, 815-826.

Ogawa, H., Wakita, M., Hasegawa, K., Kobayakawa, T., Sakai, N., Hirai, T., et al. 2005. Functional MRI detection of activation in the primary gustatory cortices in humans. Chem. Senses 30, 583-592.

Rudenga, K., Small, D. 2012. Amygdala response to sucrose consumption is inversely related to artificial sweetener use. Appetite 58, 504-507.

Rudenga, K.J., Small, D.M. 2013. Ventromedial prefrontal cortex response to concentrated sucrose reflects liking rather than sweet quality coding. Chem. Senses, 38, 585-594.

Singh, P.B., Hummel, T., Gerber, J.C., Landis, B.N., Iannilli, E. 2015. Cerebral processing of umami: A pilot study on the effects of familiarity. Brain Res.1614, 67-74.

Turkeltaub, P.E., Eden, G.F., Jones, K.M., Zeffiro, T.A. 2002. Meta-analysis of the functional neuroanatomy of single-word reading: method and validation. Neuroimage 16, 765-780.

Turkeltaub, P.E., Eickhoff, S.B., Laird, A.R., Fox, M., Wiener, M., Fox, P. 2012. Minimizing within-experiment and within-group effects in activation likelihood estimation meta-analyses. Hum. Brain Mapp. 33, 1-13.

van Rijn, I., de Graaf, C., Smeets, P.A. 2015. Tasting calories differentially affects brain activation during hunger and satiety. Behav. Brain Res. 279, 139-147.

Veldhuizen, M.G., Small, D.M. 2011. Modality-specific neural effects of selective attention to taste and odor. Chem. Senses 36, 747-760.

World Health Organization. 2006. BMI Classification. Retrieved 24 August, 2016, from http://apps.who.int/bmi/index.jsp?introPage=intro_3.html.

Yeung, A.W.K., Tanabe, H.C., Suen, J.L.K., Goto, T.K. 2016. Taste intensity modulates effective connectivity from the insular cortex to the thalamus in humans. NeuroImage 135, 214-222.

**Table S1.** Studies included in the supplementary meta-analysis reporting only ROI studies.

| Study | *n^a^* | Handed-ness^b^ | Mean age ± SD (range) | Fast time | Contrasts (stimuli)^c^ | Foci | Statistical correction^d^ | Software^e^ |
| --- | --- | --- | --- | --- | --- | --- | --- | --- |
| Cerf-Ducastel et al. (2001)^f^ | 12  (3M, 9F) | R | 23.3 ± 6.9 (20–45) | NA | Taste (aspartame/ hydrochloric acid/ quinine HCl/ NaCl) – tasteless | 14 | Uncorrected *P* < 0.01 | SPM |
| O’Doherty et al. (2002) | 8  (5M, 3F) | NA | 24.5 ± NA (18–35) | NA | Glucose – tasteless; NaCl – tasteless | 4 | Uncorrected *P* < 0.001 | SPM |
| de Araujo et al. (2003) | 11  (6M, 5F) | NA | NA | NA | Sucrose – tasteless | 8 | FWE | SPM |
| Ogawa et al. (2005) | 11  (6M, 5F) | R | 23.8 ± NA (21–31) | NA | NaCl – tasteless | 8 | FWE | SPM |
| Guest et al. (2007) | 5 (gender unidentified) | NA | NA | NA | Glucose – tasteless | 7 | FWE | SPM |
| Grabenhorst et al. (2008) | 12  (6M, 6F) | NA | NA ± NA (21–35) | 3 h | MSG – tasteless; (MSG + dilute IMP) – tasteless; (MSG + concentrate IMP) – tasteless | 7 | FWE | SPM |
| Veldhuizen et al. (2011) | 16  (16F) | R | 25.1 ± 6.4 (NA) | NA | Sucrose – tasteless | 3 | FDR | SPM |
| Rudenga et al. (2012) | 26  (10M, 16F) | NA | NA ± NA (19–38) | NA | Sucrose – tasteless | 2 | FDR | SPM |
| Nakamura et al. (2013) | 18  (9M, 9F) | R | 26.1 ± 3.2 (22–33) | 2 h | Taste (MSG/ NaCl) – tasteless | 1 | FWE | SPM |
| Rudenga et al. (2013) | 30  (12M, 18F) | NA | 24.5 ± 5.8 (NA) | NA | Sucrose – tasteless | 11 | FDR | SPM |
| Dalenberg et al. (2015) | 19  (19M) | R | 23.9 ± 2.8 (19–30) | 2 h | Taste (sucrose/ NaCl/ citric acid/ quinine HCl) – tasteless | 23 | FWE | SPM |
| Singh et al. (2015) | 10  (3M, 7F) | R | 24.9 ± NA (21–32) | NA | MSG – tasteless; NaCl – tasteless | 5 | FWE | SPM |
| van Rijn et al. (2015) | 30  (30F) | R | 22 ± 3 (18–35) | 3 h | Sweet (maltodextrin/ sucralose/ both) – tasteless | 4 | Uncorrected P < 0.001 + k > 8 | SPM |
| Yeung et al. (2016) | 34  (17M, 17F) | R | 21.3 ± 2.4 (18–27) | 2 h | NaCl – tasteless | 16 | FWE | SPM |
| Total | 242  (96M, 141F, 5?) |  |  |  |  | 113 |  |  |

^a^ F, female. M, male.

^b^ NA, not available in original paper. R, right-handed.

^c^ IMP, inosine monophosphate. MSG, monosodium glutamate.

^d^ FDR, false discovery rate. FWE, family-wise error. k, cluster size in units of contiguous voxels.

^e^ SPM, Statistical Parametric Mapping.

^f^ All studies reported results in MNI coordinate except Cerf-Ducastel et al. (2001), which reported in Talairach coordinate.

**Table S2.** Locations of signficant clusters by supplementary meta-analysis of ROI studies.

| Cluster | Brain region^a^ | Peak voxel MNI coordinates^b^ | | | Cluster size | ALE value | Contributing experiments | | | | | | |
| --- | --- | --- | --- | --- | --- | --- | --- | --- | --- | --- | --- | --- | --- |
|  |  |  |  |  |  |  | Total | | |  | Detailed breakdown | | |
|  |  | x | y | z | (mm^3^) | (×10^-2^) | No. | | %^c^ |  | Taste | No. | %^d^ |
| A | Anterior insula R | 34 | 16 | 4 | 1,880 | 2.07 | 6 | | 27 |  | Sweet | 2 | 29 |
|  |  |  |  |  |  |  |  | |  |  | Salty | 1 | 14 |
|  |  |  |  |  |  |  |  | |  |  | Umami | 3 | 60 |
|  |  |  |  |  |  |  |  | |  |  | General | 1 | 33 |
| B | Middle insula R | 40 | -4 | 10 | 3,424 | 3.19 | 10 | | 45 |  | Sweet | 1 | 14 |
|  |  |  |  |  |  |  |  | |  |  | Salty | 6 | 86 |
|  |  |  |  |  |  |  |  | |  |  | Umami | 1 | 20 |
|  |  |  |  |  |  |  |  | |  |  | General | 2 | 67 |
| C | Middle insula L | -38 | -4 | 14 | 1,352 | 1.78 | 4 | | 18 |  | Sweet | 2 | 29 |
|  |  |  |  |  |  |  |  | |  |  | Salty | 1 | 14 |
|  |  |  |  |  |  |  |  | |  |  | Umami | 1 | 20 |
| D | Mediodorsal thalamus R/ | 4 | -18 | 4 | 1,736 | 2.13 | 4 | | 18 |  | Sweet | 1 | 14 |
|  | Ventroposteromedial | *16* | *-20* | *-4* |  | 1.27 |  | |  |  | Salty | 2 | 29 |
|  | nucleus of thalamus R |  |  |  |  |  |  | |  |  | General | 1 | 33 |
| E | Postcentral gyrus R/ | 62 | -14 | 24 | 3,280 | 3.36 | 6 | | 27 |  | Sweet | 1 | 14 |
|  | Precentral gyrus R | *60* | *-2* | *30* |  | 2.72 |  |  | |  | Salty | 4 | 57 |
|  |  |  |  |  |  |  |  |  | |  | General | 1 | 33 |

Clusters were thresholded at *P* < 0.05 (cluster level family-wise error corrected for multiple comparisons).

^a^ L, left hemisphere. R, right hemisphere.

^b^ MNI, Montreal Neurological Institute. Italics indicate a peak falls under same cluster as preceding peak.

^c^ % calculated based on total experiment number (n = 22).

^d^ % calculated based on experiment number of that particular taste (sweet = 7, salty = 7, umami = 5 and general = 3).


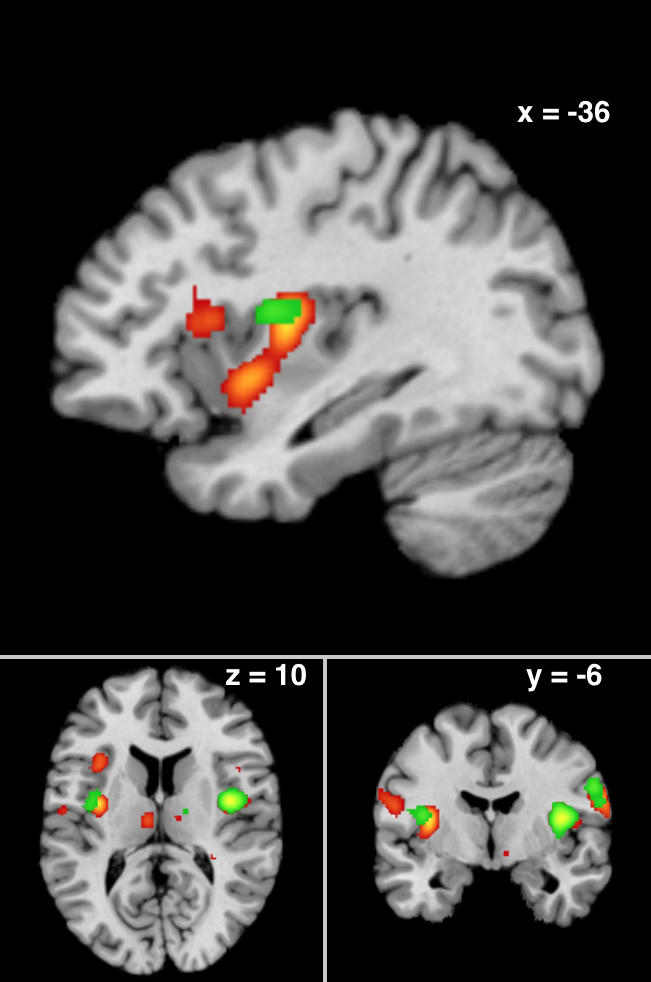


**Figure S1.** Illustration of the meta-analytic results from whole-brain studies (red) and ROI studies (green). It could be observed that ROI studies resulted in fewer clusters.
